# Safety and effectiveness of a Patient Blood Management (PBM) program in surgical patients - the study design for a multi-centre prospective epidemiologic non-inferiority trial

**DOI:** 10.1186/s12913-014-0576-3

**Published:** 2014-11-19

**Authors:** Patrick Meybohm, Dania Patricia Fischer, Christof Geisen, Markus Matthias Müller, Christian Friedrich Weber, Eva Herrmann, Björn Steffen, Erhard Seifried, Kai Zacharowski, the German PBM Study Core Group

**Affiliations:** Department of Anaesthesiology, Intensive Care Medicine and Pain Therapy, University Hospital Frankfurt, Theodor-Stern-Kai 7, 60590 Frankfurt am Main, Germany; Institute of Biostatistics and Mathematical Modelling, University Hospital Frankfurt, Theodor-Stern-Kai 7, 60590 Frankfurt am Main, Germany; German Red Cross Blood Transfusion Service Baden-Wuerttemberg – Hessen, Institute of Transfusion Medicine and Immunohematology, Sandhofstrasse 1, Frankfurt am Main, Germany; Department of Haematooncology, University Hospital Frankfurt, Frankfurt am Main, Germany

**Keywords:** Patient Blood Management, Red Blood Cell Transfusion Practice, Patient Safety, Anaemia, Clinical Outcome, Perioperative Care

## Abstract

**Background:**

Preoperative and hospital-acquired anaemia is common among surgical patients. It is associated with an increased risk of morbidity and mortality and a strong risk factor for allogeneic blood transfusions with their own inherent risks. Patient Blood Management (PBM) concepts aim to increase and preserve autologous erythrocyte volume and to optimise haemotherapy. They thus have great potential to benefit patients.

**Methods/Design:**

This prospective, multi-centre clinical trial tests the hypothesis that PBM programs are safe and effective in the care of adult surgical patients. Primary outcome is a composite endpoint of adverse events and in-hospital mortality.

**Discussion:**

This trial will determine whether the implementation of a PBM program is safe and effective in terms of clinical outcome compared to a pre-implementation cohort. This trial is registered at www.clinicaltrials.gov (NCT01820949).

## Background

Anaemia has a high prevalence among surgical patients as it is often preexisting, acquired and/or exacerbated during hospital stay [1,2]. It is associated with higher risks of morbidity and mortality and is also a major risk factor for allogeneic blood transfusions. The transfusion of allogeneic red blood cell (RBC) units is by itself also associated with increased morbidity and mortality due to infectious, immunological, pulmonary and thromboembolic complications [3-5]. Surely, adequate RBC transfusion is a life-saving medical intervention for those in need of it, but transfusion practice varies significantly when comparing hospitals and physicians, which implies that insecurity regarding adequate utilisation exists [6,7]. Additionally, serious blood supply challenges are imminent due to changing population demographics. Therefore, modifiable risk factors for transfusion such as anaemia should be targeted and a rational use of RBC units and safe clinical transfusion practice is mandatory. The World Health Organisation encourages all member states to implement Patient Blood Management (PBM) programs employing multiple combined strategies to increase and preserve autologous erythrocyte volume in order to minimise unnecessary exposure to RBC transfusions [8]. Early detection and appropriate treatment of anaemia, multidisciplinary concepts designed to maintain haemoglobin concentration, to optimise haemostasis, and to minimise blood loss shall be thrived for in an effort to improve patient outcome and ensure adequate use of scarce resources [9,10].

Several public-based PBM initiatives are in progress, e.g. in Australia, to optimise utilisation of RBC concentrates [8,11]. However, none of these initiatives is designed to provide scientific data in terms of safety issues. Thus, a large study with robust and relevant clinical endpoints is required. The object of this study is to demonstrate that the implementation of a PBM program is safe.

## Methods

### Trial design

This is a prospective multi-centre controlled epidemiologic non-inferiority trial with an approximated population of 100,000 patients undergoing surgical procedures. A patient-focused and evidence-based PBM program has been implemented in four German University Hospitals. Different time slots for control, implementation and study phases according to a non-randomised stepped wedge trial design were allocated for control, implementation and intervention periods.

### Participants

Four German university hospitals are participating in the study: Frankfurt, Bonn, Kiel, Muenster. The PBM program includes all patients undergoing surgical procedures ranging anywhere from: orthopaedic and visceral surgery, general surgery, cardiothoracic surgery, trauma, otorhinolaryngology, gynecology and obstetrics, urology, oral and maxillofacial surgery, vascular surgery, and neurosurgery to increase applicability of the evidence obtained during this trial. Exclusion criteria are: <18 years, ophthalmologic or dermatologic or outpatient surgery. If a patient has multiple hospital admissions during the study period, the first hospital stay will be analysed.

The control group will be composed of the patients treated according to the standard protocol in charge before implementation of the PBM program. A period of three months was allowed for the implementation of the PBM components. Patients treated after implementation will be allocated to the PBM cohort until July 2015.

### Management

For consistent implementation regular teaching sessions were held at the beginning of the program and are repeated every six months. The focus is on clinical practice, outcome and safety issues in order to increase knowledge on PBM and awareness of the clinical implications of anaemia and the need for alternatives to transfusion. The new program is additionally promoted through hand outs, posters, checklists and web-based information among health care providers and patients.

Our PBM program has three main pillars:

- **Preoperative optimisation of haemoglobin levels:**

**Figure 1:**
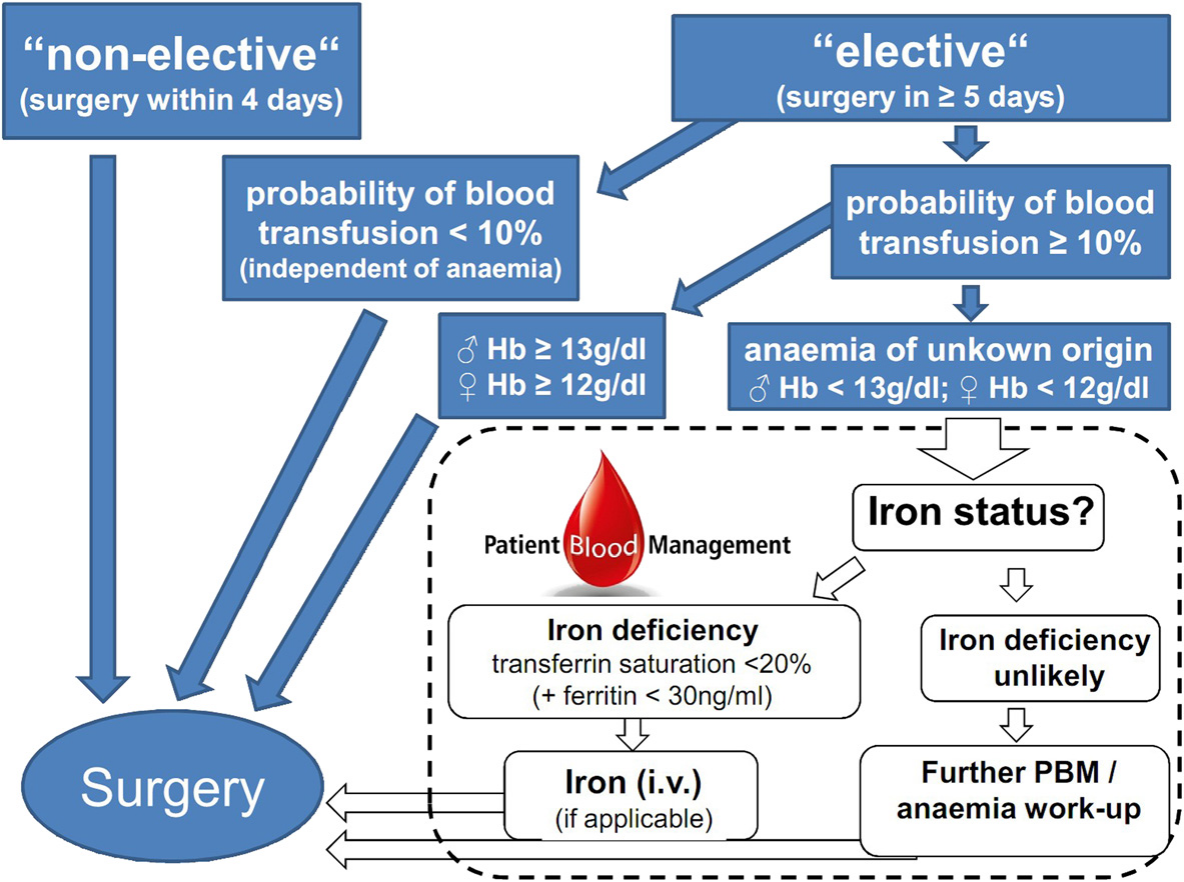
**Algorithm for the detection, evaluation and management of anaemia at the University Hospital Frankfurt.**

The preoperative detection, evaluation and therapy of anaemia diagnosis and treatment focuses specifically on those patients (aged 18 years and older) scheduled for elective surgery. Figure 1 shows the work-flow used at the University Hospital Frankfurt. Surgeries associated with a risk for transfusion >10% were identified by retrospective analysis of hospital data from 2011 with the hospital-information system Agfa ORBIS. Patients scheduled for elective surgery falling in that spectrum are screened and treated at the earliest possibility according to NATA and SABM recommendations if surgery can be postponed for at least four days (Figure 1) [12]. Iron deficiency anaemia (IDA) is treated with intravenous iron. Anaemic patients are suggested to be scheduled for both haematological and/or gastroenterological consultations for further diagnostics and optimal treatment of anaemia in cases where iron deficiency anaemia is unlikely.

b) **Standardisation of transfusion practice according to evidence-based guidelines:**

**Figure 2:**
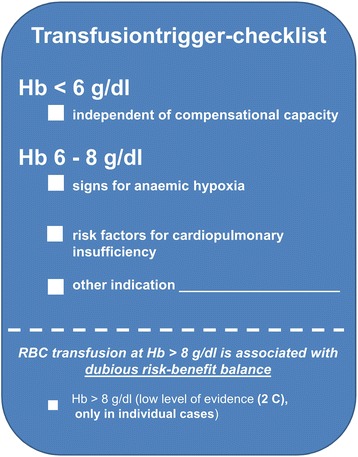
**Transfusion trigger checklist.**

**Figure 3:**
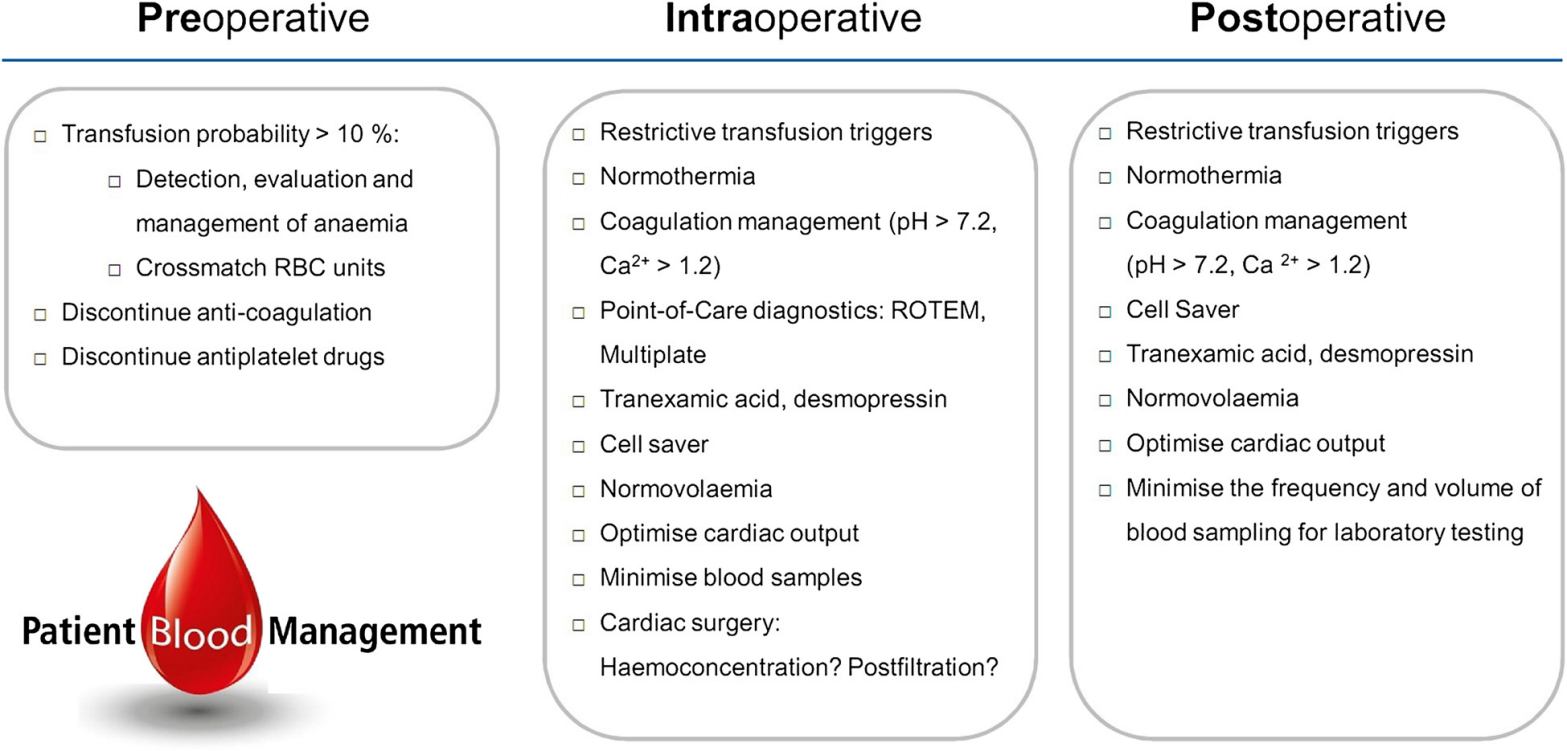
**Perioperative patient blood management checklist.**

Lectures on transfusion triggers according to the Cross-Sectional Guidelines for Therapy with Blood Components and Plasma Derivatives of the German Medical Association [13]. The strict enforcement of guideline-based transfusion triggers for surgical patients is supported by checklists attached to each RBC and/or during electronic documentation of the indication for RBC in patient’s record (Figure 2) and during the perioperative process (Figure 3).

c) **Alternatives to transfusion, and blood-sparing techniques:**

This part of the PBM program comprises a broad use of the cell saver system in defined subgroups of patients, temperature management, point-of-care diagnostics, optimised coagulation management, and restrictive blood sampling [14].

In support of the strategies above, a PBM Checklist was introduced to guide physicians through the perioperative process (Figure 3).

## Endpoints

### Primary efficacy endpoint

Primary endpoints are the following composite endpoints: in-hospital myocardial infarction, stroke, acute renal failure, death of any cause, pneumonia and sepsis until discharge from hospital in patients before and after implementation of PBM program.

### Secondary endpoints

- Length of stay on the intensive care unit
- Total hospital stay
- Quantitative utilisation of allogeneic RBC units, platelet concentrates, other blood products

### Data recording

The efficacy endpoints will be monitored by means of the hospital-information system Agfa ORBIS, where the relevant diagnoses are encoded based on the ICD-10 system. All patient-related data will be analysed electronically and anonymised. The follow-up will last until discharge from the hospital.

### Ethics

The PBM program is in accordance with state of the art transfusion guidelines and current recommendations for preoperative haemoglobin optimisation. This prospective multi-centre controlled epidemiologic trial (non-inferiority) has been approved by the ethics committees of the University Hospital Frankfurt (Ref.: 380/12) and of all participating centers. Data collection is performed anonymously. All collected data will be kept confidential. This study will be performed in accordance with the revision of the Declaration of Helsinki (2013) [15]. Since implementation will affect all hospitalised patients, information and informed consent of individual patients will not be obtained.

## Statistics

### Sample size and power calculation

According to preliminary data, the composite endpoint has an approximate incidence of 10%. A difference of 0,5% is set as the non-inferiority margin as this can be seen as the natural standard deviation. The level of significance of non-inferiority is determined be α = 2.5%. This is consistent with a two-sided 95% confidence range of 1 + α = 95% for the difference in the composite endpoint. A power of 1 + β = 80% is sought. As the number of patients treated in each of the four university hospitals vary, an exact sample size calculation is not possible to this date. However, we expect approximately 100,000 participating patients.

### Statistical analysis

The primary aim is to prove non-inferiority of the intervention (PBM) cohort when compared with the control cohort stratified by center. The primary composite endpoint will be registered electronically by analysis of ICD-10 diagnoses and will be exported to a data base. It will be analysed with a one-sided Mantel-Haenszel test with significance level of α =2.5% for the odds ratio resulting in H0: OR ≥ OR* vs. H1 OR < OR* with OR* being derived from the incidence in the control cohort and an incidence rate of the PBM cohort which is increased by the non-inferiority margin of 0.5%. As secondary analysis, we will analyse two-sided 95% confidence interval for the difference in the frequency of the composite safety endpoint as well as for the odds ratios and the single components of the composite endpoint. Additionally, the influence of covariates, e.g. the calendar year, concomitant diseases, the asynchrony of the study periods at different sites will be assessed statistically. Should there be a significant influence on the incidence of the composite endpoint, the Mantel-Haenszel test will be supplemented with a respective multivariate approach accounting for covariates.

Secondary endpoints will be compared with a two-sided Wilcoxon-Mann–Whitney test using a significance level of α =5%. A subgroup analysis will assess if there is some bias from not excluding patients with multiple hospital stays for surgical intervention during the study period. A further subgroup analysis will assess if there is a difference of safety between a) elective patients who will receive preoperative haemoglobin optimisation compared to b) anemic patients who will not undergo preoperative haemoglobin optimisation due to failed postponement of surgery in case of surgical urgency or non-elective surgery, respectively.

### Economic evaluation

A cost-utility analysis is being conducted. Resource use data such as blood products transfused, inpatient days by ward type, surgery, medications, complications and their treatment has been integrated into the statistical analysis. The average cost in each group will be calculated, and from this the incremental cost-effectiveness ratio will be derived.

### Clinical study monitoring

An Independent Data Monitoring and Safety Committee will analyse the data on a regular basis. If the implementation of the PBM program will result in a 5% rise of the primary efficacy endpoint compared to the control cohort encompassing all recruited patients from all centers, the IDMC can recommend early termination of the study. Final decision for early termination will be done by the principle investigators.

## Discussion

While transfusion of cellular blood components is a life-saving intervention, over-transfusion can be associated with short and long-term hazards. Therefore risks and benefits of transfusions should be weighed individually in every patient and modifiable risk factors for transfusion such as anaemia should be prevented and treated accordingly [5,9,16,17]. An effective, patient-focused PBM program could help to save a scarce resource and to improve patient outcome and safety [18,19]. In the last ten years, different aspects of a PBM program have been implemented into clinical practice reducing the utilisation of allogeneic red blood cell transfusions and improved outcome [20-24].

Nonetheless, there remains uncertainty about the safety and effectiveness of PBM. Both fear of insufficient tissue oxygenation following a restrictive transfusion trigger and delay of surgery due to preoperative optimisation are the main concerns prevailing.

This study aims to investigate whether or not a PBM program is non-inferior in terms of patient outcome compared to a pre-implementation cohort. The acceptance of the PBM program among physicians will determine the success of this study.

In conclusion, this prospective, multi-centre trial intends to determine whether or not the implementation of a PBM program is safe and effective in about 100.000 hospitalised patients undergoing surgery.

## Trial status and conclusion

This trial is registered at www.clinicaltrials.gov (Identifier: NCT01820949).

The Patient Blood Management program has been implemented in the four university hospitals of Frankfurt, Bonn, Kiel and Muenster.

Despite the complex nature of implementing new clinical standards, this trial has proceeded successfully. The experience and data from this study should help to design and implement fitting PBM programs in other hospitals.

## Abbreviations

IDA: Iron-deficiency anaemia
PBM: Patient Blood Management
RBC: Red blood cells

## References

[1] Musallam KM, Tamim HM, Richards T, Spahn DR, Rosendaal FR, Habbal A, Khreiss M, Dahdaleh FS, Khavandi K, Sfeir PM, Soweid A, Hoballah JJ, Taher AT, Jamali FR (2011). Preoperative anaemia and postoperative outcomes in non-cardiac surgery: a retrospective cohort study. Lancet.

[2] Koch CG, Li L, Sun Z, Hixson ED, Tang A, Phillips SC, Blackstone EH, Henderson JM (2013). Hospital-acquired anemia: Prevalence, outcomes, and healthcare implications. J Hosp Med.

[3] Marik PE, Corwin HL (2008). Efficacy of red blood cell transfusion in the critically ill: a systematic review of the literature. Crit Care Med.

[4] Shander A, Goodnough LT (2009). Why an alternative to blood transfusion?. Crit Care Clin.

[5] Vamvakas EC, Blajchman MA (2009). Transfusion-related mortality: the ongoing risks of allogeneic blood transfusion and the available strategies for their prevention. Blood.

[6] Frank SM, Savage WJ, Rothschild JA, Rivers RJ, Ness PM, Paul SL, Ulatowski JA (2012). Variability in blood and blood component utilization as assessed by an anesthesia information management system. Anesthesiology.

[7] Bennett-Guerrero E, Zhao Y, O’Brien SM, Ferguson TB, Peterson ED, Gammie JS, Song HK (2010). Variation in use of blood transfusion in coronary artery bypass graft surgery. JAMA.

[8] Farmer SL, Towler SC, Leahy MF, Hofmann A (2013). Drivers for change: Western Australia Patient Blood Management Program (WA PBMP), World Health Assembly (WHA) and Advisory Committee on Blood Safety and Availability (ACBSA). Best Pract Res Clin Anaesthesiol.

[9] Spahn DR, Goodnough LT (2013). Alternatives to blood transfusion. Lancet.

[10] Gombotz H, Zacharowski K, Spahn DR (2013). Patient Blood Management: Individual treatment to reduce and prevent anaemia.

[11] Shander A, Van Aken H, Colomina MJ, Gombotz H, Hofmann A, Krauspe R, Lasocki S, Richards T, Slappendel R, Spahn DR (2012). Patient blood management in Europe. Br J Anaesth.

[12] Shander A, Goodnough LT, Javidroozi M, Auerbach M, Carson J, Ershler WB, Ghiglione M, Glaspy J, Lew I (2014). Iron Deficiency Anemia-Bridging the Knowledge and Practice Gap. Transfus Med Rev.

[13] German Medical Association (BÄK) (2008). Cross-sectional Guidelines for Therapy with Blood Components and Plasma Derivatives.

[14] Fischer D, Zacharowski KD, Meybohm P (2014). Savoring every drop - Vampire or Mosquito?. Crit Care.

[15] World Medical A (2013). World Medical Association Declaration of Helsinki: ethical principles for medical research involving human subjects. Jama.

[16] Glance LG, Dick AW, Mukamel DB, Fleming FJ, Zollo RA, Wissler R, Salloum R, Meredith UW, Osler TM (2011). Association between intraoperative blood transfusion and mortality and morbidity in patients undergoing noncardiac surgery. Anesthesiology.

[17] Acheson AG, Brookes MJ, Spahn DR (2012). Effects of allogeneic red blood cell transfusions on clinical outcomes in patients undergoing colorectal cancer surgery: a systematic review and meta-analysis. Ann Surg.

[18] Gombotz H (2011). Patient blood management is key before elective surgery. Lancet.

[19] Spahn DR, Moch H, Hofmann A, Isbister JP (2008). Patient blood management: the pragmatic solution for the problems with blood transfusions. Anesthesiology.

[20] Brevig J, McDonald J, Zelinka ES, Gallagher T, Jin R, Grunkemeier GL (2009). Blood transfusion reduction in cardiac surgery: multidisciplinary approach at a community hospital. Ann Thorac Surg.

[21] Kotze A, Carter LA, Scally AJ (2012). Effect of a patient blood management programme on preoperative anaemia, transfusion rate, and outcome after primary hip or knee arthroplasty: a quality improvement cycle. Br J Anaesth.

[22] Emmert MY, Salzberg SP, Theusinger OM, Felix C, Plass A, Hoerstrup SP, Falk V, Gruenenfelder J (2011). How good patient blood management leads to excellent outcomes in Jehovah’s witness patients undergoing cardiac surgery. Interact Cardiovasc Thorac Surg.

[23] Hofmann A, Farmer S, Towler SC (2012). Strategies to preempt and reduce the use of blood products: an Australian perspective. Curr Opin Anaesthesiol.

[24] Theusinger OM, Felix C, Spahn DR (2012). Strategies to reduce the use of blood products: a European perspective. Curr Opin Anaesthesiol.

